# Epidemiological features and survival outcomes in patients with malignant pulmonary blastoma: a US population-based analysis

**DOI:** 10.1186/s12885-020-07323-0

**Published:** 2020-08-26

**Authors:** Xiang Bu, Jing Liu, Linyan Wei, Xiqiang Wang, Mingwei Chen

**Affiliations:** 1 Department of Respiratory and Critical Care Medicine, the First Affiliated Hospital of Xi’an Jiaotong University, Xi’an, Shaanxi Province China; 2 Key Laboratory of Molecular Cardiology, Xi’an, Shaanxi Province China; 3 Department of Cardiovascular Medicine, the First Affiliated Hospital of Xi’an Jiaotong University, Xi’an, Shaanxi Province China

**Keywords:** Pulmonary blastoma, Long-term prognosis, SEER database, Competing-risk model

## Abstract

**Background:**

Pulmonary blastoma (PB) is a rare lung primary malignancy with poorly understood risk factors and prognosis. We sought to investigate the epidemiologic features and long-term outcomes of PB.

**Methods:**

A population-based cohort study was conducted to quantify the death risk of PB patients. All subjects diagnosed with malignant PB from 1988 to 2016 were screened from the Surveillance, Epidemiology and End Results database. Cox regression model of all-cause death and competing risk analysis of cause-specific death were performed.

**Results:**

We identified 177 PB patients with a median survival of 108 months. The 5 and 10-year survival rate in all PB patients were 58.2 and 48.5%, as well as the 5 and 10-year disease-specific mortality were 33.5 and 38.6%. No sex or race disparities in incidence and prognosis was observed. The death risk of PB was significantly associated with age at diagnosis, clinical stage, histologic subtype and surgery treatment (*p*<0.01). On multivariable regression analyses, older age, regional stage and no surgery predicted higher risk of both all-cause and disease-specific death in PB patients.

**Conclusion:**

We described the epidemiological characteristics of PB and identified its prognostic factors that were independently associated with worse clinical outcome.

## Background

Pulmonary blastoma (PB) is a rare subtype of human lung tumors accounting for approximately 0.25–0.5% of primary pulmonary malignancies. There are only about a few hundreds of cases reported worldwide since the first description by Barnett and Barnard in 1945 [1–3]. These tumors morphologically resembling embryonal lung structure were historically described under a uniform medical term until distinct entities were recognized [4]. Childhood PB, also referred as pleuropulmonary blastoma (PPB) [5], occurs almost exclusively in children and adolescents and is characterized by localized-regional evolution with some cases exhibiting more aggressive and metastasizing properties [6, 7]. On contrast, Adult-onset PB is more common in middle-aged people and typically presents with non-specific clinical manifestations similar to lung cancer. It is further classified into two subtypes: monophasic PB, which is also called well-differentiated fetal adenocarcinoma (WDFA), and classic biphasic PB (CBPB) containing tissue of both fetal adenocarcinoma (typically of low grade) and primitive mesenchymal stroma [8, 9]. The existence of partially-overlapping genetic abnormalities in PPB, CBPB and WDFA has been proved [10], and the original pathological grouping and histological characteristics of these three subtypes are similar and coherent. Due to the rarity of PB, there are few researches exploring the long-term outcome of these populations. Most previous studies are case reports and literature reviews focusing on a small number of subjects, the results are ambiguous and even controversial. The aims of our study were to describe the epidemiological features of malignant PB in detail and to investigate the independent prognostic factors for PB patients.

## Methods

### Study population

The Surveillance, Epidemiology and End Results (SEER) database (https://seer.cancer.gov/), a publicly available cancer database covering 34.6% of the US population, was applied to retrieve patients diagnosed with malignant PB between 1988 and 2016, using National Cancer Institute’s SEER*Stat software (version 8.3.5). The diagnosis of PB has to be histologically confirmed by surgery or lymph node biopsy. Histology codes (International Classification of Disease for Oncology, third edition, ICD-O-3) 8972/3, 8973/3 and ICD 8333/3 were used for the identification of all CBPB, PPB and WDFA cases, respectively. The International Classification of Diseases-10 (ICD-10) codes were used to identify the underlying causes of death. PB patients with unavailable cancer-specific data and vital status were excluded. Informed consent was not required for the analysis of the data from SEER.

### Clinically applicable predictors and primary outcome

The primary focus of this study was given to the potential predictors of overall survival (OS) and disease-specific survival (DSS) in patients with malignant PB. Predictors were specified based on the availability in clinical practice of PB and the published literatures [2, 11–13]. Age at diagnosis was divided into 3 groups: ≤14 years, 15–64 years, and ≥ 65 years. Race was classified into white, black and other (American Indian/AK Native, Asian/Pacific Islander). The year of diagnosis was categorized into two periods: 1988–2006 and 2007–2016. Clinical variables included anatomical laterality (left, right and others), primary site (upper lobe, lower lobe, and other sites), histological subtype (CBPB, PPB and WDFA), clinical stage (localized, regional and distant), surgery status (yes/no) and presence of second or more primary malignancies (yes/no). “Others” in anatomical laterality was defined as “bilateral sites” or “unspecified site”. Other primary sites included main bronchus, pleura, subcutaneous tissue and other soft tissue, and overlapping lesion of lung, heart, mediastinum and pleura. Tumor stage was described as “localized” if it is entirely confined to the original organ, “regional” if it extends to regional lymph nodes and/or surrounding organs or tissues, and “distant” if it has metastasized to distant organs or lymph nodes according to SEER staging system. We chose not to include tumor grade as an indicator for two reasons: firstly, this information was unknown for almost 60% of the cases; secondly, PPB and CBPB are generally not graded and WDFA is by definition grade I (although high grade fetal adenocarcinoma also has been described) in the clinical practice. Follow-up time was defined as the time from diagnosis to the date of death, last contact or end of the study period (31 December 2016), whichever occurred first. Subjects with any missing data relevant to the outcome were excluded from our study in order to perform a complete case analysis.

### Statistical analysis

The distributions of all baseline data were summarized by calculating the frequencies for categorical variables, which were further analyzed by chi-square to determine statistical significance. The median follow-up time was evaluated using the reverse Kaplan–Meier method. Hazard ratios (HRs) and 95% confidence intervals (95% CIs) for mortality associated with various potential predictors were calculated using Cox univariate analysis. For multivariate analysis, Cox proportional hazards regression modeling was adopted to identify the predictors independently associated with death risk by adjusting for a large set of covariates. R program (Version 3.6.3, R core team) was used to perform statistical analysis and make figures according to a priori defined study protocol. All tests were 2-sided, and statistical significance was set as *p*-value of < 0.05.

### Sensitivity analysis

In addition to the primary analysis, sensitivity analysis was conducted to evaluate the robustness of our findings. We applied competing-risk model to test, under careful consideration of the competing risk events of our interest events, how the conclusions would be affected. The commonly used endpoint target of competitive risk analysis was the cumulative incidence function (CIF). Crude cumulative mortality was calculated and plotted for disease-specific death and death from other causes among PB patients. Additionally, stratified analyses by predictors with statistical significance were performed. Competing-risk model was completed using the R package ‘cmprsk’ (R Foundation for Statistical Computing, Vienna, Austria) [14, 15].

## Results

### Baseline characteristics of study population

The demographic and clinical characteristics of all PB patients were shown in Table 1, as well as the parameters variation for 3 different histological subtypes. A total of 177 identified PB cases between 1988 and 2016 were eligible to be included in the study, of whom 67 cases were younger than 15 years old at the time of diagnosis (age ≤ 14, 37.9%). 55.4% of the patients were female and 73.4% were white. The number of patients diagnosed during the near decade accounted for more than half of all cases (54.2% in 2007–2016 vs. 45.8% in 1987–2006). The incidences of PB on different anatomical lateralities were 42.4, 55.3, and 2.3% for left side, right side and others, respectively. The most common primary site was the upper lung lobe (40.7%). The majority of patients were diagnosed in earlier stages of the tumor (local or regional, 80.2%), which was an important reason for a large proportion of patients to receive surgery (86.4%). One interesting phenomenon we observed was that 22.6% (40 out of 177) of PB patients were accompanied by other primary malignant tumors, and PB was not the first primary cancer in 62.5%(25 out of 40) of these patients with multiple primary cancer. With regard to histological subtypes of PB, CBPB (51.5%) was the most frequent, followed by PPB (31.6%) and WDFA (16.9%). Three subtypes were significantly different in the age of onset (p<0.001). CBPB occurred in all ages and contributed most of the elderly cases (age ≥ 65, 30 in 36). In contrast, WDFA was much more common in middle-aged group and PPB occurred exclusively in children as we mentioned before. The incidence of three subtypes all showed a slight female preponderance, while no significant difference in sex distribution was found among three groups. Most cases of PPB and WDFA were diagnosed between 2007 and 2016, while most cases of CBPB were diagnosed before 2007(p<0.001). PB was not the first primary cancer in 17.6% (16 out of 91) of CBPB cases, 3.6% (2 out of 56) of PPB cases and 23.3% (7 out of 30) of WDFA cases (*p* = 0.013). Other characteristics showed no significant difference among three histological subtypes.

**Table 1:** Baseline characteristics of PB patients in different stratifications of histologic subtype

Patient Characteristics	All patients Cases (%)	Histologic Subtype (Cases, %)			
		CBPB (91, 51.5%)	PPB (56, 31.6%)	WDFA (30, 16.9%)	*p* value
Age at diagnosis (years)					<0.001 ***
≤ 14	67(37.9%)	11 (12.1%)	55 (98.2%)	1 (3.3%)	
15–64	74 (41.8%)	50 (54.9%)	0 (0.0%)	24 (80.0%)	
≥ 65	36 (20.3%)	30 (33.0%)	1 (1.8%)	5 (16.7%)	
Sex					0.841
Male	79 (44.6%)	42 (46.2%)	25 (44.6%)	12 (40.0%)	
Female	98 (55.4%)	49 (53.8%)	31 (55.4%)	18 (60.0%)	
Race/Ethnicity					0.083
White	130 (73.4%)	69 (75.8%)	44 (78.6%)	17 (56.7%)	
Black	36 (20.3%)	19 (20.9%)	7 (12.5%)	10 (33.3%)	
Other	11 (6.2%)	3 (3.3%)	5 (8.9%)	3 (10.0%)	
Year of diagnosis					<0.001 ***
1988–2006	81 (45.8%)	57 (62.6%)	17 (30.4%)	7 (23.3%)	
2007–2016	96 (54.2%)	34 (37.4%)	39 (69.6%)	23 (76.7%)	
Anatomical Laterality					0.418
Left	75 (42.4%)	35 (38.5%)	29 (51.8%)	11 (36.7%)	
Right	98 (55.3%)	53 (58.2%)	26 (46.4%)	19 (63.3%)	
Others	4 (2.3%)	3 (3.3%)	1 (1.8%)	0 (0.0%)	
Primary Site					0.085
Upper lobe	72 (40.7%)	36 (39.6%)	19 (33.9%)	17 (56.7%)	
Lower lobe	50 (28.2%)	29 (31.9%)	13 (23.2%)	8 (26.7%)	
Other sites	55 (31.3%)	26 (28.6%)	24 (42.9%)	5 (16.7%)	
Clinical Stage					0.495
Localized	92 (52.0%)	43 (47.3%)	29 (51.8%)	20 (66.7%)	
Regional	50 (28.2%)	28 (30.8%)	16 (28.6%)	6 (20.0%)	
Distant	35 (19.8%)	20 (22.0%)	11 (19.6%)	4 (13.3%)	
Surgery					0.108
Yes	153 (86.4%)	74 (81.3%)	51 (91.1%)	28 (93.3%)	
No	24 (13.6%)	17 (18.7%)	5 (8.9%)	2 (6.7%)	
Only Primary					0.080
Yes	137 (77.4%)	67 (73.6%)	49 (87.5%)	21 (70.0%)	
No	40 (22.6%)	24 (26.4%)	7 (12.5%)	9 (30.0%)	
First Primary					0.013 *
Yes	152 (85.9%)	75 (82.4%)	54 (96.4%)	23 (76.7%)	
No	25 (14.1%)	16 (17.6%)	2 (3.6%)	7 (23.3%)	

A total of 177 patients with malignant PB were stratified by different histologic subtype, and the demographic and clinical characteristics were summarized by calculating the frequencies for categorical variables. P value was calculated by the chi-square analysis. All tests were 2-sided, and statistical significance was set as *p*-value of < 0.05. * and *** indicated *p*<0.05, *p*<0.001 respectively (R program, Version 3.6.3, R core team)

*PB* pulmonary blastoma, *PPB* pleuropulmonary blastoma, *WDFA* well-differentiated fetal adenocarcinoma, *CBPB* classic biphasic PB

### Incidence and risk of all-cause death

**Fig. 1:**
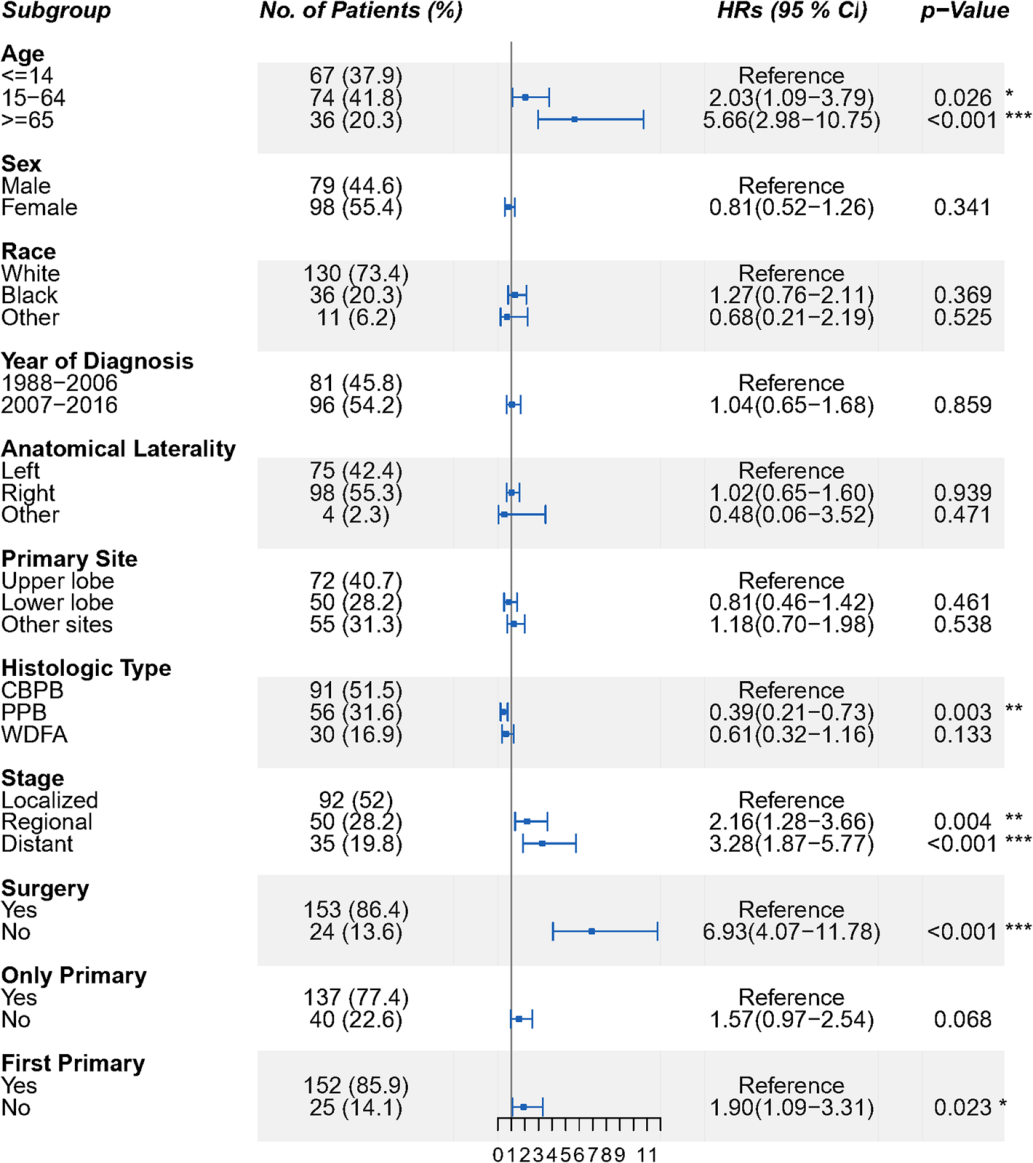
Forest plot of HR for all-cause death in PB patients. A total of 177 patients with malignant PB were stratified by different factors. HRs and 95% CIs for all-cause death in different stratifications were calculated using Cox models, with the first subgroup as reference. The *p* values were for the difference between subgroups in each stratification. All tests were 2-sided, and statistical significance was set as *p*-value of < 0.05. *, ** and *** indicated *p*<0.05, *p*<0.01 and *p*<0.001 respectively (R program, Version 3.6.3, R core team). PB, pulmonary blastoma; PPB, pleuropulmonary blastoma; WDFA, well-differentiated fetal adenocarcinoma; CBPB, classic biphasic PB; HR, hazard ratio; CI, confidence interval

**Fig. 2:**
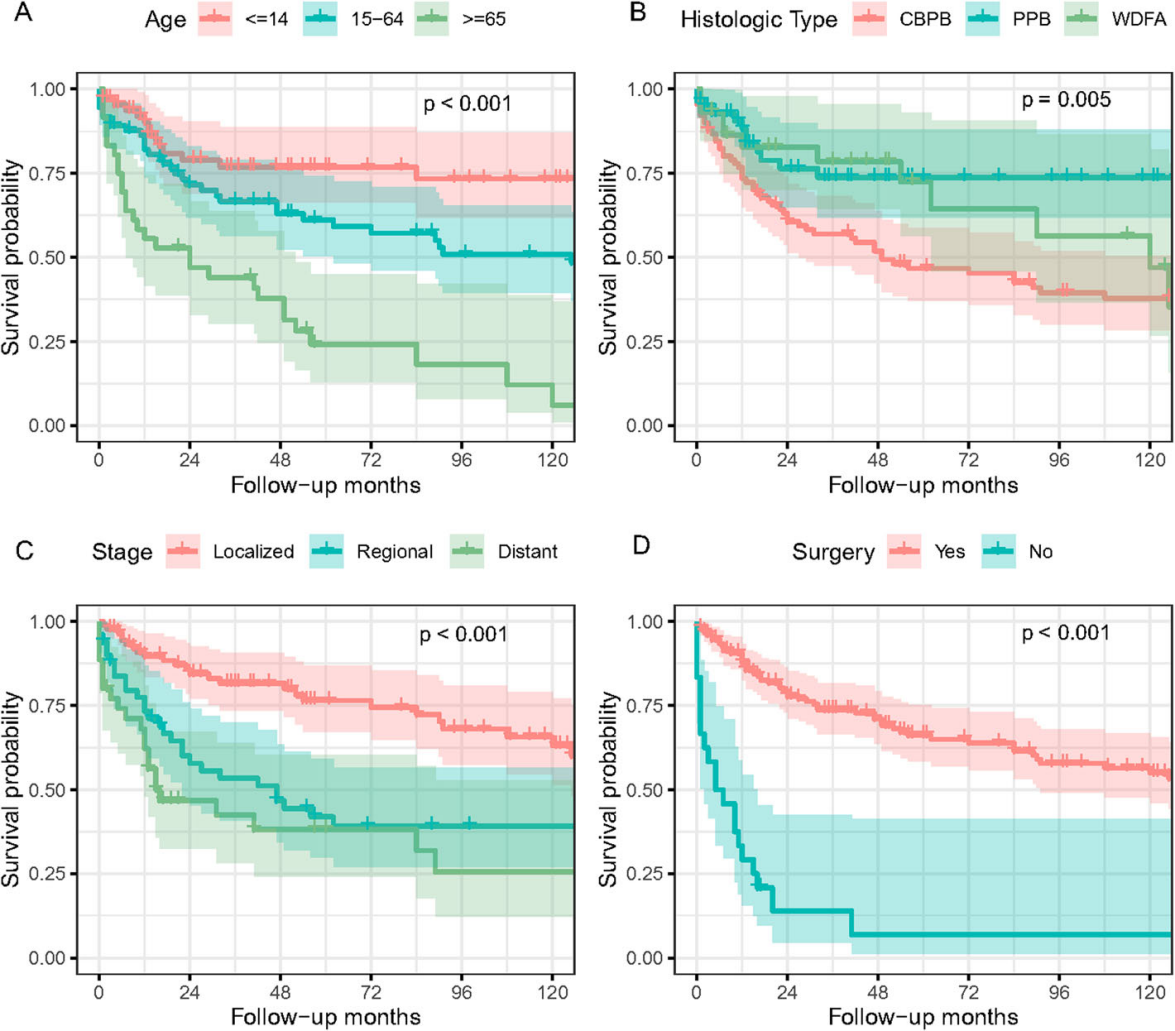
Kaplan–Meier survival plots for PB patients. Kaplan–Meier plots for overall survival of PB patients stratified by (**a**) age at diagnosis, (**b**) histologic subtype, (**c**) clinical stage and (**d**) surgery or not. The p values for comparison of the cumulative survival probability in different stratifications were calculated using Log Rank (Mantel-Cox) test (R program, Version 3.6.3, R core team). PB, pulmonary blastoma; PPB, pleuropulmonary blastoma; WDFA, well-differentiated fetal adenocarcinoma; CBPB, classic biphasic PB

There were 78 patients of all-cause death (44.1%) during the follow-up period. The associations between individual prognostic factors and OS among all PB patients were presented in Figs. 1 and 2. Median follow-up time for all PB patients was 108 months, with 39 cases (22.0%) having more than 10 years’ follow-up. As shown in Fig. 1, the OS was most strongly related to age, with hazard ratios (HRs) as high as 2.03 (95% CI: 1.09–3.79) and 5.66 (95% CI: 2.98–10.75) for patients aged 15–64 and ≥ 65 compared to patients aged ≤14, respectively. Patients who were 65 years and older had the worst clinical outcome, and almost half of them died within 2 years (Fig. 2a, *p*<0.001). Patients with regional or distant tumor stage had dramatically increased risk of all-cause death (regional, HR: 2.16, 95% CI:1.28–3.66; distant, HR: 3.28, 95% CI: 1.87–5.77) compared to those with localized stage. The 5-year OS for patients with localized stage was more than 75%, while for patients with regional or distant stage it was less than 40% (Fig. 2c, *p*<0.001). As expected, no surgical treatment portended worse outcome with a quite higher death risk (HR: 6.93, 95% CI: 4.07–11.78). Nearly 70% of non-operated patients died in the first year, but the 5-year OS for operated patients was above 60%. (Fig. 2d, *p*<0.001).

As to the survival difference in three histological subtypes of PB, PPB patients showed a significant reduction in all-cause death compared to CBPB patients (HR: 0.39, 95% CI: 0.21–0.73), and their OS stabilized at around 75% after a 30-month follow-up, after which all survivors achieved long-term survival. WDFA patients had a lower risk of death compared to CBPB patients, but the differences showed no statistical significance (Fig. 2b, *p* = 0.005). Moreover, patients with multiple malignant cancers suffered a higher all-cause death risk than those only with PB, although no significant difference was observed between two groups (HR:1.57, 95% CI: 0.21–0.73, *p* = 0.068) (Supplement Fig. 1A). No significant decrease of all-cause death was observed in patients who were diagnosed between 2007 and 2016 compared to those diagnosed between 1988 and 2006, despite of the great advances in medical care during this decade. Other factors like sex and race of the patients, as well as the primary site and anatomical laterality of the tumor, were not observed to be significantly associated with the clinical outcome of PB (Supplement Fig. 1B-F).

### Cumulative incidence of disease-specific death

**Fig. 3:**
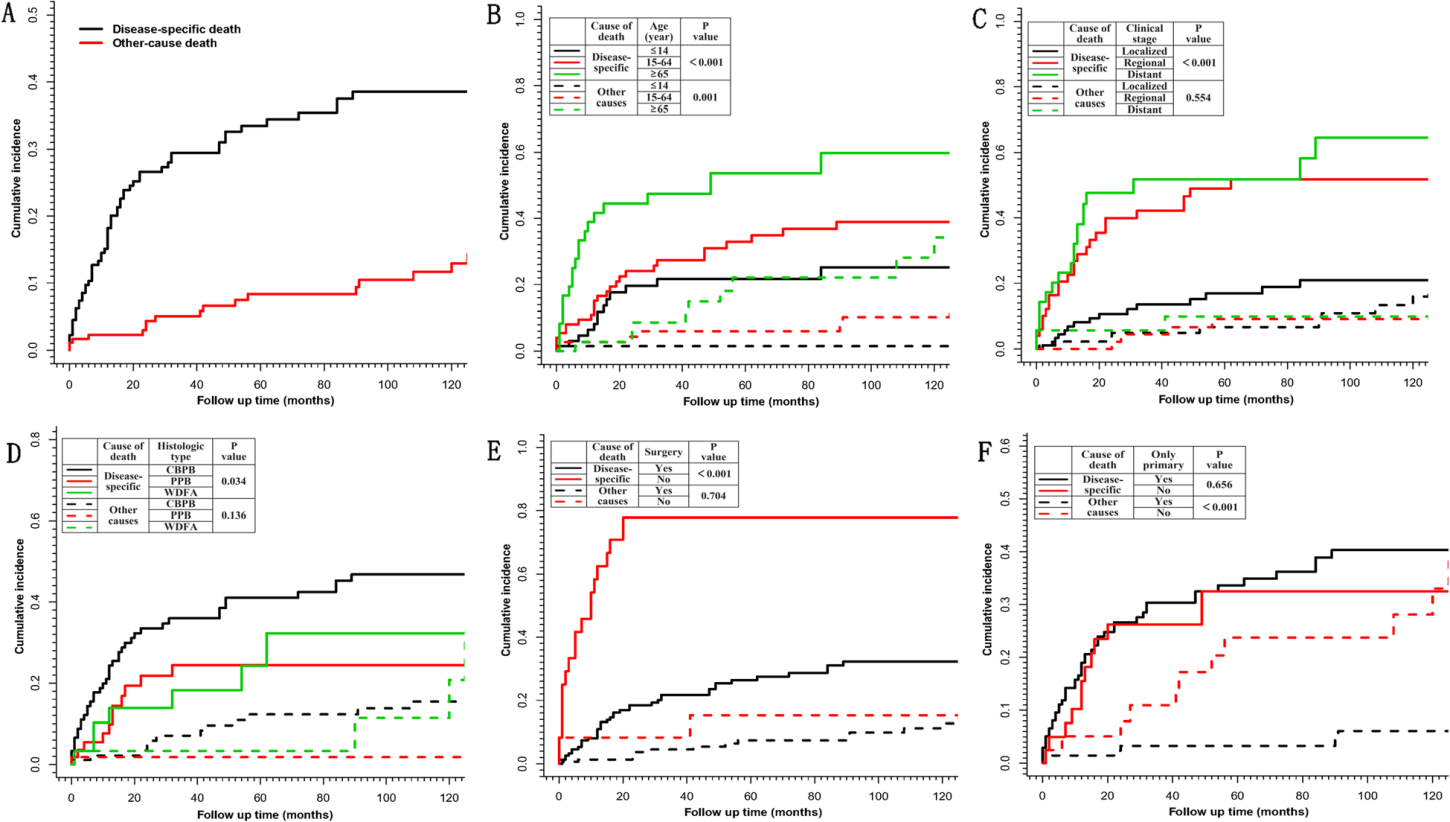
Cumulative incidence of cause-specific death for PB patients. The cumulative incidence of cause-specific death for (**a**) overall patients and stratified patients by (**b**) age at diagnosis, (**c**) clinical stage, (**d**) histologic subtype, (**e**) surgery or not and (**f**) the number of primary malignancy. The p values for comparison of the cumulative incidence functions according to different stratification are based on Gray’s test (R program, Version 3.6.3, R core team). PB, pulmonary blastoma; PPB, pleuropulmonary blastoma; WDFA, well-differentiated fetal adenocarcinoma; CBPB, classic biphasic PB

The cumulative mortality for various causes of death among all patients were illustrated in Fig. 3a. PB caused by far the main mortality, with the majority of disease-specific deaths occurring within 30 months. During this time, the cumulative disease-specific mortality rose rapidly to nearly 30%, whereafter gradually stabilized below 40%. The 5 and 10-year disease-specific mortality of PB was 33.5 and 38.6%, respectively. By contrast, the other-caused cumulative mortality showed a relatively flat upward trend with the extension of follow-up time, and the 10-year mortality caused by other reasons remained below 15%.

#### Disease-specific mortality stratified by age and clinical stage

The cumulative incidences of disease-specific death and other-caused death among PB patients in different stratifications were illustrated respectively. There were 60 cases of disease-specific death (33.9%) and 18 cases of other-cause death (10.2%) during the follow-up period. Stratified by age groups (Fig. 3b), patients aged 65 years or older had the highest cumulative disease-specific mortality and other-caused mortality compared with patients in other groups. Other-caused mortality sharply increased with age at diagnosis(p<0.001) and length of follow-up, but the disease-specific mortality between three age groups showed no statistical difference (*p* = 0.066). The association between clinical stage and disease-specific mortality was strong, and the regional or distant stage presented a much worse outcome with quite higher disease-specific mortality compared with localized stage (p<0.001). Strikingly, almost half of the patients with distant stage died of this tumor within 20 months (Fig. 3c).

#### Disease-specific mortality stratified by histological subtype and other factors

Figure 3d demonstrated the disease-specific mortality among patients with different histological subtypes. The association between histological subtype and clinical outcomes of PB patients was still pronounced when it comes to the risk of disease-specific death (p<0.05). Few patients with PPB died from causes other than this disease, and almost 80% of them could achieve long-term survival after 30 months’ follow up. The disease-specific mortality and other-cause mortality of patients with CBPB were higher than those of patients with PPB or WDFA. The disease-specific mortality of CBPB patients sharply increased to 40% within the first 3 years, and then gradually approached 50% in the ten-year’s follow up. The death rate from other causes in CBPB was close to 10%, accounting for a quarter of the overall cumulative mortality in the fifth year. As to the effect evaluation of surgery (Fig. 3e), it was observed that the cumulative incidences of disease-specific death in patients without surgery was close to 80% within 20 months, which was significantly higher than that in operated patients (p<0.001). In addition, to examine the association between co-existing primary cancers and cause of death, we stratified the cohort into two groups with or without a second or more primary cancers (Fig. 3f). The findings suggested some new changes in the trend of the association. Although the cumulative incidences of disease-specific death between two groups showed no statistic difference, the main cause of death in patients without other primary malignancy was PB itself, while nearly half of the death of patients with multiple malignant cancers was caused by other reasons other than PB itself (p<0.001).

### Independent predictors of all-cause death and disease-specific death

Multivariate analysis was further performed to investigate the independent prognostic factors for survival among PB patients. All above-mentioned predictors with statistical significance in univariate analysis, including age stratification, histological subtype, clinical stage, surgery treatment, and one or more primary malignancies, were included into Cox proportional hazards regression model or proportional subdistribution hazards regression model, respectively. Two multivariate analysis models yielded nearly identical results. Age stratification, clinical stage and surgery treatment turned out to be independently associated with the all-caused death and disease-specific death in PB patients, while the statistical correlation between histological subtype and the survival of PB patients was no longer significant. The calculated effect sizes and *p* values from two analysis models were reported and compared in Table 2.

**Table 2:** Multivariate Cox regression analysis and competing risks analysis

Clinical predictors	Multivariate Cox analysis^1,#^		Competing risks analysis^2,#^	
	HR (95% CI)	*P* value	HR (95% CI)	*P* value
Age at diagnosis				
≤ 14	Reference		Reference	
15–64	2.191 (1.172–4.098)	0.014 *	1.50 (0.766–2.95)	0.24
≥ 65	5.192 (2.690–10.021)	<0.001 ***	3.63 (1.738–7.60)	<0.001 ***
Clinical Stage				
Localized	Reference		Reference	
Regional	2.458 (1.448–4.171)	0.001 **	3.30 (1.857–5.87)	<0.001 ***
Distant	1.898 (0.986–3.653)	0.054	2.21 (0.983–4.96)	0.055
Surgery				
Yes	Reference		Reference	
No	5.139 (2.630–10.042)	<0.001 ***	4.29 (1.992–9.25)	<0.001 ***

^1^ Using Cox proportional hazards regression model

^2^ Using proportional subdistribution hazards regression Model

#Adjusted for predictors with statistical significance in univariate analysis (age at diagnosis, clinical stage, histologic subtype, number of primary malignancy and surgery or not)

Multivariate Cox regression analysis was for all-cause death and competing risks analysis was for disease-specific death in PB patients. The p values for Cox model were calculated using Log Rank (Mantel-Cox) test, and for competing risk model were based on Gray’s test. HR, hazard ratio; CI, confidence interval. Statistical significance was set as *p*-value of < 0.05. *, ** and *** indicated *p*<0.05, *p*<0.01 and *p*<0.001 respectively (R program, Version 3.6.3, R core team)

## Discussion

Although PB has been known for over 70 years, its prognostic factors remain largely unknown. Limited evidence suggests that age of onset, gender, anatomical location, tumor size and stage, histologic subtype, comorbidities and metastasis status and surgical resection may be associated with different outcome, but these results warrant further validation [2, 6–9]. PB is previously reported to be an aggressive tumor with relatively poor prognosis. Some previous literature mentions that two-thirds of PB patients die within 2 years of diagnosis, only 16 and 8% survive 5 and 10 years post diagnosis, respectively [13, 16, 17]. But in our study, nearly half of the PB patients achieved long-term survival, the 5 and 10-year survival rate in all PB patients were 58.2 and 48.5%, even 40% of patients with metastatic PB achieved long-term survival over 5 years. It could be seen that the survival rate of PB in our study was quite higher than that in previous reports [18]. In addition, we found a slight female preponderance in the incidence of PB, and female patients were more common in all three histological subtypes. Whereas, the clinical outcomes between two gender groups indicated no significant difference. Nearly a quarter of PB patients were associated with other malignancies, which had not been reported before. Considering the previously published papers were almost case reports and literature reviews with small sample size, our study was more informative.

The three subtypes of PB, CBPB, PPB and WDFA, are reported to be distinguished on morphological, immunohistochemical and radiographical and clinical outcome grounds. The World Health Organization (WHO) classification of lung tumors in 2004 qualifies CBPB as lung sarcomatoid carcinoma, PPB as pulmonary soft tissue tumor and WDFA as a lung adenocarcinoma variant [4]. Patients with CBPB generally present with common symptoms of lung cancer and larger diameter tumors [19]. WDFA often radiographically manifests as peripheral asymptomatic nodules with mixed solid and cystic components [20]. The biological characteristics of PPB are unique, and the tumors often undergo a transition from cystic to solid based on different subtypes and disease progresses, of which type I is associated with better prognosis and type III has the worst prognosis [21]. The 5-year survival for CBPB was reported about 15% versus 62% for PPB and about 75% for WDFA [2, 5, 11, 20]. It was worth noting that the 5-year survival rate between different histological subtypes in our study did not show such huge disparity despite statistical differences (Fig. 2b), and the mortality gap among three subtypes was even smaller when it comes to disease-specific death (Fig. 3d). Further multivariate analysis also indicated that histological subtype was not an independent predictor of prognosis in PB patients.

As our results suggested, peak ages of onset in three subtypes were quite different. PPB occurred almost exclusively in children aged 14 years or younger, which was why the OS and DSS of PPB patients were almost identical. On the contrary, CBPB occurred in all ages and mostly in middle-aged and old patients, and the chances of dying from other factors other than PB itself were increasing dramatically with age (Fig. 2b). Other-caused deaths even accounted for 30% of overall deaths in patients 65 years or older at diagnosis (9 of 30). Undoubtedly, the difference in mortality between different histological subtypes could be influenced by the uneven distribution of the number of patients at different ages. The impact of PB on OS was much more potent in the older cohort. New molecular data indicates that PB patients share some overlapping molecular profiles, and DICER1 mutations are found to be important drivers and are likely to be associated with the later presentation of both CBPB and WDFA, as well as PPB [10]. The similarities and differences among three subtypes of PB should be explored further.

Surgical excision is regarded as the optimal treatment choice for well-localized mass and regional disease, 86.4% of the patients in our study performed surgery. Consistent with previous reports [22, 23], surgery treatment significantly prolonged the survival time of PB patients, and the cumulative incidences of disease-specific death in operated patients was much lower than that in non-operated patients. Expanded resection plus lymph node dissection is the preferred method of PB treatment, and the specific range of operations should be customized according to individual clinical features. Postoperative radiochemotherapy can be performed when lymph node metastasis or surrounding tissue involvement is observed. However, it’s reported that only a few cases were sensitive to radiotherapy [24]; Cutler et al. summarized the clinical outcome of 468 patients who underwent postoperative chemotherapy and found that the effect of single or combined medication was not satisfactory, and the median survival of these patients was only 14.7 months [25]. Some scholars believe that the survival time of PB is mainly related to the degree of resection and the prognosis of patients with complete resection is better. While some other scholars think that, the effect of PB surgery largely relies on the differentiation of mesenchymal components. Patients with immature, undifferentiated and embryonic-like tumor tissues have the better prognosis. Because there are few cases of continuous long-term follow-up before and after surgery, the optimal therapeutic regimen of PB needs to be further explored.

In this registry-based cohort study, SEER database, a large population-based resource, was applied to provide valuable information of these low-incidence malignancies. To our knowledge, this study had the largest number of subjects among all researches conducted so far on the long-term clinical outcome of patients with malignant PB. Our results filled some previous gaps in terms of epidemiology of PB, as well as added new evidence to current controversial issues about the prognosis of PB patients. Another highlight of the study was that we used two different statistical methods to analyze the overall survival and disease-specific mortality of PB patients during various follow-up period. As we all know, there are multiple endpoint events in prospective observational cohort study, and if one event may affect the probability of another event or completely hinder its occurrence, they will be competitive risk events for each other. The standard Kaplan–Meier analyses reflect mortality from the event of interest without the consideration of competing events. This approach of treating failures from competing events as censored will lead to an overestimation of the absolute risk of the event of interest and is less clinically relevant [26]. Therefore, we applied the competitive risk model, an analytical method designed for the survival data with multiple potential outcomes, to calculate the disease-specific mortality in a condition of retaining the underlying risk set for patients who died due to competing causes of death. As we mentioned above, similar findings were observed in the competitive risk model analysis. And the independent prognostic factors for PB predicted by two different statistical models were the same, which further showed the robustness of our results.

Our study had several limitations. First, this was a retrospective study based on administrative information from the SEER database. Therefore, clinical variables such as tumor morphology, chemoradiotherapy information, complications and medication use were lacking. In addition, details from the surgery procedures and preoperative TNM-classification were not available. Second, as with any other retrospective study, we could not exclude the possibility of residual or unmeasured confounding. Third, although SEER is designed to approximate the national distribution of cancer characteristics by collecting cancer incidence data from population-based cancer registries in the USA, it is derived from 18 states and covers only 34.6% of the U.S. population, which may lead to over- or under-representation of certain hospital types and limit its generalizability to other population. Another limitation of this study was its small sample size due to the low incidence rate of PB, resulting in the compromise in quality of estimates. Nevertheless, the unique strengths in this study were the preciseness of statistical analyses and the long follow-up time, which partially increased the power of test.

## Conclusion

In conclusion, older age, biphasic tumors (CBPB), initial presence of metastasis (stage of distant) and not receiving surgery were identified to be closely associated with an unfavorable prognosis of PB. The independent predictors of both all-cause death and disease-specific death in PB patients were age stratification, clinical stage and surgery treatment. In a word, our study filled some previous gaps in terms of PB epidemiology, provided new evidence to current controversial issues about the prognosis of this rare lung cancer, and would be helpful to guide the prognosis estimation of PB patients.

## Supplementary information

**Additional file 1: Supplement Figure 1.** Kaplan–Meier survival plots for PB patients. Kaplan–Meier plots of cumulative survival for PB patients stratified by (A) the number of primary malignancy, (B) sex, (C) race/ethnicity, (D) year of diagnosis, (E) anatomical laterality and (F) primary site of the tumor. The *p* values for comparison of the cumulative survival in different stratifications are calculated using Log Rank (Mantel-Cox) test (R program, Version 3.6.3, R core team).

## Data Availability

The datasets generated and/or analysed during the current study are available in the [SEER] repository, [https://seer.cancer.gov/].

## Abbreviations

PB: Pulmonary blastoma
PPB: Pleuropulmonary blastoma
WDFA: Well-differentiated fetal adenocarcinoma
CBPB: Classic biphasic PB
SEER: The Surveillance, Epidemiology and End Results database
OS: Overall survival
DSS: Disease-specific survival
HR: Hazard ratio
CI: Confidence interval
CIF: Cumulative incidence function
WHO: World Health Organization
