# Supplementary figures and images for: Epidemiological features and survival outcomes in patients with malignant pulmonary blastoma: a US population-based analysis
Source: BMC Cancer. 2020 Aug 26;20:811. doi: 10.1186/s12885-020-07323-0 (PMC7449001; doi:10.1186/s12885-020-07323-0)

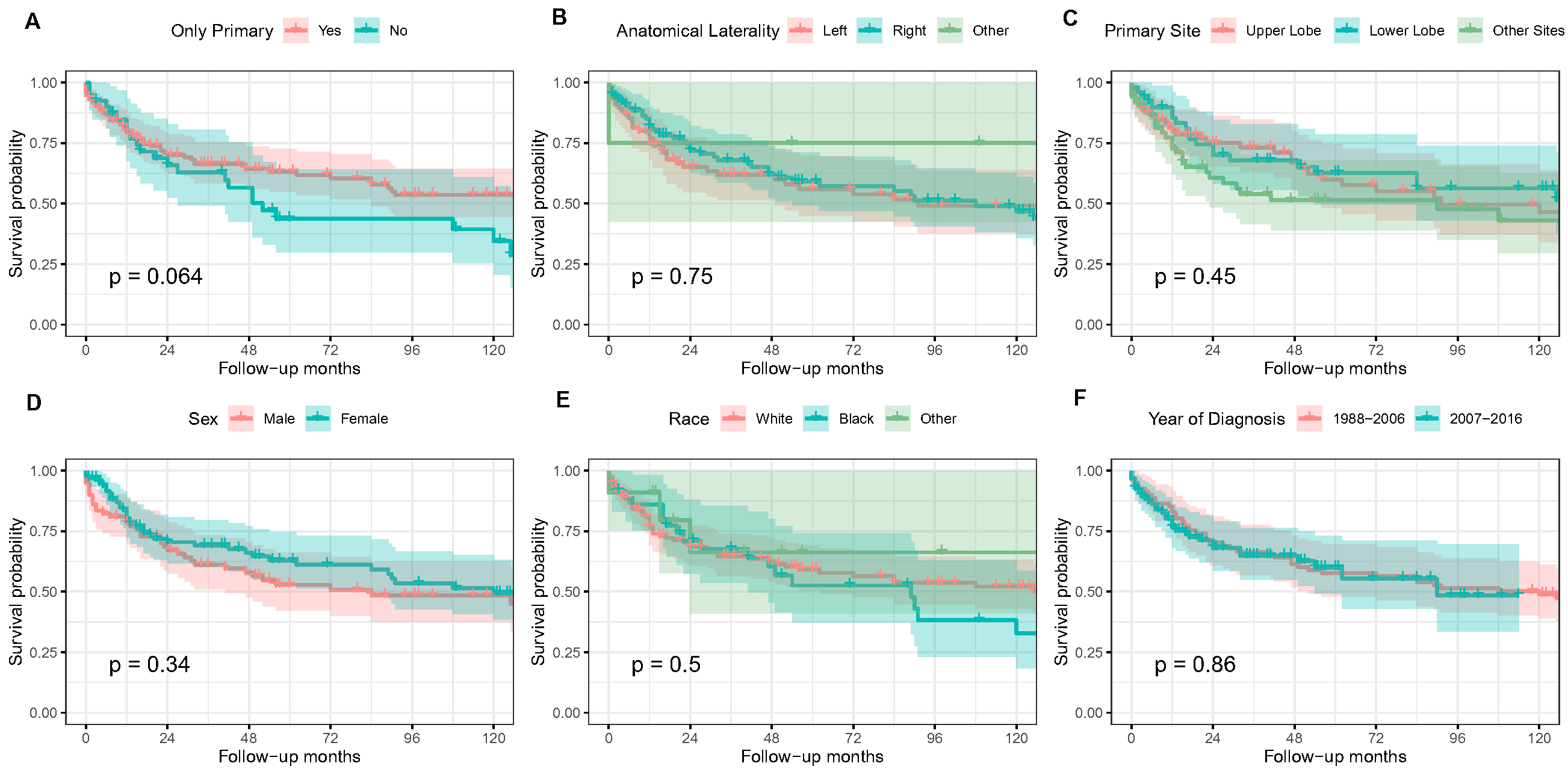

Supplement: Supplementary file 1 — Additional file 1: Supplement Figure 1. Kaplan–Meier survival plots for PB patients. Kaplan–Meier plots of cumulative survival for PB patients stratified by (A) the number of primary malignancy, (B) sex, (C) race/ethnicity, (D) year of diagnosis, (E) anatomical laterality and (F) primary site of the tumor. The p values for comparison of the cumulative survival in different stratifications are calculated using Log Rank (Mantel-Cox) test (R program, Version 3.6.3, R core team). [file 12885_2020_7323_MOESM1_ESM.tiff]
